# Coca: The History and Medical Significance of an Ancient Andean Tradition

**DOI:** 10.1155/2016/4048764

**Published:** 2016-04-07

**Authors:** Amy Sue Biondich, Jeremy David Joslin

**Affiliations:** Department of Emergency Medicine, SUNY Upstate Medical University, 550 E. Genesee Street, Suite 103, Syracuse, NY 13202, USA

## Abstract

Coca leaf products are an integral part of the lives of the Andean peoples from both a cultural and traditional medicine perspective. Coca is also the whole plant from which cocaine is derived. Coca products are thought to be a panacea for health troubles in regions of South America. This review will examine the toxicology of whole coca and will also look at medicinal applications of this plant, past, present, and future.

## 1. Introduction

Coca is an indigenous plant of South America with numerous alkaloid components, the most well-known one of which is the psychoactive component, cocaine. Its leaves have been a staple in the Andean lifestyle for thousands of years. Strong interest in coca use has existed in the anthropological world for decades. Areas of study have not only attempted to understand traditional use during the Incan empire and coca's role in folk medicine, but also focused on factors that influence the ability of this native tradition to continue in the face of increasingly strict regulations of coca production. Efforts have been made to understand the long-term Andean use of coca, with subsequent research examining the scientific basis behind local beliefs. This review will examine the history of coca production, its toxicity profile, and its varied uses throughout the centuries. It is important to note that although coca continues to be valuable to native Andean people for religious and cultural reasons, travelers to these regions need to be aware that there is a paucity of medical data supporting the safe use of coca. The astute toxicologist should maintain an understanding of the cultural pressures experienced by contemporary travelers.

## 2. Coca Species

The Aymara people are an indigenous population of the Andes and Altiplano regions of South America. “Khoka” is an Aymara word that means “the tree.” This is the origin for our modern usage of “coca” [1]. The coca shrub is indigenous to South America, Mexico, Indonesia, and the West Indies. It is one of the oldest cultivated plants of South America [2]. Cultivated coca plants belong to two distinct species of the genus* Erythroxylum* (family Erythroxylaceae):* Erythroxylum coca* Lam. and* Erythroxylum novogranatense* (Morris) Hieron [3]. There are two varieties to be found within each of the cultivated species of coca.* E. coca* Lam. var.* coca* is also known as “Bolivian” or “Huanuco” coca. This is the best-known variety and continues to be widely cultivated throughout the Andean region for both legal uses and the illegal production of cocaine [2]. Other varieties are found in smaller areas and are mainly cultivated for coca chewing or other traditional uses by local peoples [4].

Cocaine is the principal alkaloid found in the cultivated varieties of coca. Cocaine was first isolated in 1860 by Dr. Albert Nieman from leaves of cultivated cocaine [5]. It is also the most studied and discussed alkaloid in the scientific literature.* E. coca* var.* coca*, the most widely cultivated variety, has been found to contain approximately 0.6% cocaine in its dried leaves [6]. However, there are a number of other biologically active alkaloids that have been studied. The four cultivated* Erythroxylum* varieties contain eighteen alkaloids, belonging to the tropanes, pyrrolidines, and pyridines [2].

An extensive review by Novák et al. details the previously studied biological activity of several of the alkaloids found in coca. Compounds reviewed include cinnamoylcocaine, benzoylecgonine, methylecgonine, pseudotropine, benzoyltropine, tropacocaine, *α*- and *β*-truxilline, hygrine, and cuscohygrine. All compounds were found to be considerably less toxic than cocaine with a lack of the euphoric effects that cocaine is known to have. The reader is referred to the articles by Jenkins et al. and Rivier for identification and quantitation of the alkaloids found in whole coca [7, 8]. However, in agreement with Novák et al. conclusion, the overall effect gained from using whole coca products may be in fact from the sum total of all plant constituents, rather than just cocaine alone. In addition, coca leaves include calories, carbohydrates, minerals, and vitamins which could also lend a source of energy and nutrients to its user.

## 3. Toxicity and Other Medical Concerns

The bulk of toxicology research is based on the pure isolate, cocaine, from coca leaves; therefore the extrapolation to the toxicity of whole coca is somewhat limited. However, of the studied biologically active alkaloids in coca, cocaine is the limiting factor in reaching a toxic dose. The physiologic effects of other alkaloids are considerably less than that of cocaine [2]. A toxicity study performed on rats found that the alkaloids benzoylecgonine and ecgonine methyl ester did not produce toxic manifestations when infused at rates that were found to be toxic for cocaine [9]. In fact, 30- and 60-fold higher doses of these alkaloids were necessary to produce any neurobehavioral changes, which ultimately proved to be mild. Benzoylecgonine did not prove to be lethal even when given in doses greater than 100 times that of cocaine [9].

In comparison to modern usage of cocaine isolates, the amount of the drug used by native peoples was and remains quite low with an estimated 60 grams of coca leaf chewed per day [1]. A coca leaf typically contains between 0.1 and 0.9% cocaine [10]. Another study that examined over 3,000 coca users found that mine workers, typically the largest consumers of coca products, chew roughly 13 ounces (368.5 grams) per week which is a similar consumption rate to previously referenced studies [11]. This would mean that an average user would extract approximately 3.9 net grams of the alkaloids contained in coca per week. This would give a maximal total dosage of roughly 200–300 mg per a 24-hour period. The actual dosage is likely much less as the user will not absorb 100% of the alkaloid. The amount of cocaine to be found in coca tea products has also been assessed. Depending on the origin of the tea bags (Bolivia versus Peru), after exhaustive extraction, an average of 4.86–5.11 mg of cocaine was found per tea bag [8]. An investigation conducted in Bolivia found that after chewing 30 g of coca leaves, whole blood cocaine levels reach around 98 ng [12].

In contrast, there is a large difference in what an average cocaine user is exposed to in modern times. A “line” of cocaine bought on the street contains an estimated 20–50 mg of cocaine hydrochloride [13]. Modern users may administer cocaine through several routes: insufflation, intravenous injection, smoking, ingestion, or mucosal application. The half-life of cocaine is 0.7–1.5 hours [14, 15]. Cocaine is rapidly cleared from the plasma but can be detected in the brain, ocular fluid, and liver for 8 hours after initial usage [16]. Following single doses of cocaine, plasma concentrations typically average 200,000–400,000 ng or 4890 ng in whole blood [17, 18]. The whole blood concentration of cocaine is almost 50 times greater after using the pure isolate in comparison to chewing whole coca. Plasma half-life and blood concentrations are both known to be dose dependent.

The estimated minimum lethal dose of cocaine is 1.2 g [19]. However, there have been case studies where individuals have died from as little as 30 mg applied to mucous membranes, and there are also addicts who may tolerate up to 5 g daily [20, 21]. The LD50 of cocaine or the dose determined to be lethal in 50% of the test population has been found to be 95.1 mg/kg in mice. For whole coca, the LD50 is considered to be 3450 mg/kg [22]. In rats, the accepted LD100 is 100 mg/kg [22]. Extrapolating these numbers to humans is not entirely generalizable; however if this data is applied to the standard 70 kg human, it can be seen that these would be very large amounts of coca.

The pharmacology of cocaine is complex with several organ systems affected simultaneously by the drug. Systems affected by acute and chronic use of cocaine include psychological, neurological, renal, cardiac, pulmonary, gastrointestinal, obstetrical, and otolaryngological ones. As mentioned in the toxicology data, medical and physiologic effects in the literature concentrate on the pure isolate cocaine. As outlined above, coca is quite different from cocaine, including common dosages and safety profiles. Local use patterns of coca are also quite different from that of individuals using the purer isolate, cocaine. Because of this, it is thought that cocaine studies have limited generalizability to coca. It is interesting to note that coca tea ingestion has resulted in a positive urine assay for cocaine metabolite [23]. It is worth noting that there is no evidence in the literature to support habitual whole coca use causing addiction or withdrawal physiology in contrast to that of cocaine users.

Cocaine serum concentrations are determined by the following: (1) dose; (2) route of administration; (3) binding to plasma proteins; and (4) rate of metabolism [13]. Most pharmacologic effects of cocaine are neuroexcitatory in nature, with resultant agitation, combativeness, hyperactivity, and seizures [24]. Cocaine is sought out for its euphoric effects, but it can also cause agitation, anxiety, panic, and psychosis [25]. Both hemorrhagic and ischemic stroke are known consequences of cocaine use [16]. Because of the potent vasoconstrictive effects on vascular smooth muscle, cocaine leads to widespread vascular dysfunction. This vasoconstriction and its anesthetic properties are the mechanisms behind the widespread effects of cocaine on multiorgan systems. In addition to vasoconstrictive effects, cocaine is also an inhibitor of tPA. This is the mechanism for accelerated atherosclerosis in users [16]. Cocaine also acts as a sodium channel blocker which can result in dysrhythmias [16]. Physiologic consequences of cocaine in a medical context are well described in the literature.

## 4. Historical Background

The antiquity of coca use is well established through archaeological studies in South and Central America. Analysis of mummified human remains from Northern Chile indicates the use of coca as early as 1000 BC [26]. These records establish that the use of coca has been ongoing for over 3,000 years in the native peoples of the Andes.

Under Incan rule, coca was used for numerous purposes, including ritual, social, and physiological uses [27]. In 1532, a Spanish expedition conquered the Inca. The Spanish conquerors then attempt to eradicate the use of coca in the native cultures [28]. However, after the elimination of coca proved unsuccessful, the Spanish then decided to exploit coca growth. Subsequently, coca use became more widespread throughout the former Incan empire, and the custom of giving agricultural workers a ration of coca leaves along with their daily wage began [28, 29]. Coca use continues to be a daily staple in the life of many Andean workers.

Interest in coca was prompted in the nineteenth century in Europe and the United States as discussed in an article by Dr. Weil [30]. Mantegazza spent years practicing medicine in Peru. Based on his experience with the local peoples, he became convinced of the widespread benefits of coca including reduction of fatigue, improvements to one's mood, and even the increase of sexual vigor [31]. Editorials on coca made their way into the British Medical Journal [32], and even writings of Sigmund Freud reflected the belief that whole coca could be a psychological cure-all [33]. The interest in whole coca quickly diminished after the isolation of cocaine. The bulk of research has been conducted on the cocaine isolate rather than the fourteen other alkaloids known to be contained in the coca plant [34]. This has led to a paucity of scientific knowledge of whole coca along with any potential medicinal effects that it may have.

## 5. Use in Contemporary Medicine

The Euro-American enthusiasm for coca quickly cooled in the early twentieth century with the increasing concerns of the addictive properties and poor side effect profiles witnessed with the use of cocaine [29]. Coca importation has since been banned in the United States.

### 5.1. Gastrointestinal Symptom Treatment

The South American Indians continue to rely on coca as a medicinal remedy in addition to its general usages for stimulant and social purposes [35]. One of the more traditional roles that coca plays in Andean life is for the relief of gastrointestinal upset. Coca leaf tea is taken to combat stomach pain, intestinal spasm, nausea, indigestion, and even constipation and diarrhea [30]. It is essentially viewed as a comprehensive remedy that restores balance to the digestive system. Coca is masticated or held in the mouth for relief of painful oral sores and also to aid in the healing of oral lesions [30]. Similarly, this plant is used for toothaches as well.

### 5.2. Environmental Stress Treatment

One of the usages of coca that continues to intrigue the medical community is as a remedy for dealing with the stresses of life at high altitude [36]. Given the strong belief in the inherent energizing effects of coca, this could explain the benefit of using this leaf in the high stress environment of high altitude. Coca is also believed to possess properties that help the user withstand hypoxia, cold, and hunger. A series of experiments in the 1970s aimed to test the hypothesis of whether coca chewing was associated with the feeling of warmth. Hanna and Little found that hand and foot temperatures were lowered in Andean people who used coca [37, 38]. Although the thermal difference between control and experimental subjects was not great, this small difference could be advantageous in decreasing the amount of heat loss in extreme environments.

### 5.3. Hunger Treatment

Coca chewing is thought to decrease the feeling of hunger in Andean peoples. Further investigation of this phenomenon has discovered that coca has effects on glucose homeostasis. Chewing coca leaves has been found to elevate blood glucose above the fasting level [39]. This finding led Bolton to believe that coca has a fundamental metabolic function for those Indians with difficulties in glucose homeostasis. This finding of elevated glucose after coca chewing also lends scientific credibility to the native belief that coca staves off hunger pains [40].

### 5.4. Altitude Illness Treatment

As the drive to understand coca's place in Andean culture increased, there was a flurry of work to pin down the mechanism in which the benefits of taking coca existed. Specific to the realm of high altitude medicine, coca chewers report less head pain and dizziness associated with working at high altitudes [41, 42]. Fuchs et al. used available ethnographic and physiologic data to propose a working hypothesis of how coca chewing was aiding individuals who lived and worked in high altitude settings [43]. Through collation of data, it was shown that the percentage of native peoples using coca increases with altitude. This generalization extends to other cultural groups and the female gender, which typically do not exhibit the high acceptance and use of coca as Quechua or other high altitude cultural groups.

Although previous work had shown that physiological adaptations to cold may be enhanced by coca use [37, 38], Fuchs et al. noted that the frequency of coca use does not necessarily correlate with climate. Mineworkers use coca extensively, and yet this is an environment not solely defined by frigid temperatures or high altitude [44]. An interesting new hypothesis was that coca's beneficial properties may lie in the mitigation of effects of hypoxia. Polycythemia is a standard response to the sustained hypoxia of high altitude environments [45]. However, controversy now exists as to whether this beneficial physiologic change can become a maladaptation when taken to the extreme. A detrimental effect of polycythemia is increased blood viscosity, thought to be the mechanism in chronic mountain sickness. Ultimately, Fuchs et al. proposed that the alkaloids present in whole leaf coca pharmacologically inhibit the stimulation of excessive red blood cell production caused by hypoxia. The decreased polycythemic stress decreases the symptoms of mountain sickness and alters the mechanisms by which the body adjusts to the high altitude environment [43].

The belief in coca as an uplifting agent for those individuals working in a high altitude environment is not without its naysayers. Many have argued that it is merely a “spurious correlation” between coca chewing and high altitude [46]. Other theories propose that coca use is thought to be a function of how “Indian” a given society is or that it is used for its general stimulant properties rather than as a specific treatment for symptoms related to high altitude [46]. It remains to be seen if any consensus will be arrived at in the anthropological quest to integrate traditional patterns of coca consumption within a biological or physiological framework.

## 6. Conclusions

Anthropologists continue to examine patterns of coca use among natives to the Andean regions of South America in order to assist in the preservation of this ancient practice. Possession of coca is illegal in many countries due to one of its products, cocaine. International treatises have called for the destruction of all coca farming. Barring Bolivia and Peru, coca is now banned, even for indigenous use. Despite this ongoing controversy, some practitioners who span the cultural and medical divide have proposed the use of coca for contemporary ailments. Due to consumer demand for more natural forms of treatment, folk medicine may be considered in modern pharmacologic contexts. Possible uses could include gastrointestinal conditions, fast-acting antidepressant, treatment for acute mountain sickness, as an energizer for persons involved in physical labor, and symptomatic treatment of toothache and oral lesions [30]. Because the known toxicity and pharmacologic profiles almost exclusively pertain to the pure isolate of cocaine, and not whole coca, controlled trials would need to be undertaken prior to any recommendation for coca in a medical context.

Although most of the theories produced by these scientists are based on observational and not experimental data, merit is to be given for attempting to understand a custom that has been ongoing for thousands of years. The pursuit of continued research from a physiologic or medical perspective will add to the respect of cultural traditions by trying them to give them a venue in which to thrive in our modern world.
